# ‘Prescribing’ psychotropic medication to our rivers and estuaries

**DOI:** 10.1192/bjb.2018.72

**Published:** 2019-08

**Authors:** Alex T. Ford, Helena Herrera

**Affiliations:** 1 University of Portsmouth

**Keywords:** Antianxiety drugs, antidepressants, antipsychotics, anxiety disorders, depressive disorders

## Abstract

**Summary:**

The influence of pharmaceuticals on the environment is an increasing concern among environmental toxicologists. It is known that their growing use is leading to detectable levels in wastewater, conceivably causing harm to aquatic ecosystems. Psychotropic medication is one such group of substances, particularly affecting high-income countries. While these drugs have a clear place in therapy, there is debate around the risk/benefit ratio in patients with mild mental health problems. Therefore, it is necessary to evaluate the wider implications as risks could extend beyond the individual to non-target organisms, particularly those in rivers and estuaries.

**Declaration of interest:**

None.

## Psychotropic drug use patterns

Adequate psychiatric service provision is central to health and quality of life, and the use of psychotropic medication can be invaluable in the treatment and management of mental health problems. The uses of these drugs have been evident since their introduction, and they have a well-established place in clinical practice. However, within the medical fields, there has been a debate in recent years about the over-reliance on drugs such as antidepressants, antianxiety drugs and antipsychotics.^1^^–^^4^ Data from a number of countries have indicated rapid rises in prescriptions, with as many as 10% of the population taking such medications, particularly in high-income countries such as the UK.^3^ Within the field of environmental toxicology, the effects of these drugs have recently started to generate some interest too.

## Pharmaceuticals as emergent pollutants

Currently, pharmaceuticals mainly enter the environment through wastewater after their excretion, either in their original form or as metabolites. This has long been known,^5^^,^^6^ with the first reports of these substances entering the environment dating back to the 1960s.^7^ However, sewage treatment plant processes do not adequately remove pharmaceuticals. With the sheer number of different medications being prescribed and the inability of even quite modern sewage treatment processes to fully break them down, it could be argued that our aquatic life is bathing in a soup of multiple drugs.

Toxicity levels of pharmaceuticals in the environment do not necessarily relate to high concentrations, but to their constant low-level discharge, persistence in ecosystems and highly active biological functions. In this way, pharmaceuticals that are found in relatively low concentrations could be extremely potent and very persistent, and able to significantly affect non-target organisms. For example, low concentrations of antidepressants and other psychotropic drugs can cause disruption to the normal functioning of aquatic organisms.^8^

The concept of environmental relevance, therefore, becomes important. For example, selective serotonin reuptake inhibitors, selective serotonin–noradrenaline reuptake inhibitors, serotonin antagonist reuptake inhibitors, tricyclic antidepressants and benzodiazepines have all been detected in urbanised waterways, mostly in the ng/L range, but also in concentrations up to μg/L.^7^ What is particularly starting to interest scientists is that these antidepressants can cause disruption to the normal functioning of aquatic life in laboratory experiments at low concentrations. The uptake of these compounds appears to be highly dependent on the organism's mode of feeding.^9^ In terms of presence in the environment, for example, diazepam has been found in all matrices – wastewater, surface, ground and drinking water, soils, bio-solids and tissues^10^ – and in concentrations as high as 10 ng/L in rivers and potable water.^11^

Pharmaceuticals are not present in isolation in the environment, and the widespread high use of a wide variety of drugs leads to multiple substances being found together, in a situation where synergistic or antagonistic effects can occur.^7^ In addition, exposure of some non-target organisms to these substances takes place for the entirety of their life cycle.^12^ Currently, pharmaceuticals and their active metabolites are globally considered to be an important emergent group of pollutants that are intrinsically bioactive, causing adverse drug reactions and previously undocumented effects on non-target organisms.^7^

## The effects of psychotropic drugs on the aquatic environment

The notion that drugs prescribed to humans might be affecting wildlife first came to light in the 1990s, when scientists highlighted that natural and synthetic oestrogens from contraceptive pills and hormone replacement therapy in wastewater effluent could feminise fish at very low concentrations.^13^ Growing incidences of reproductive and other abnormalities developed into an interdisciplinary field, linking human and environmental toxicology to the study of endocrine-disrupting chemicals. As improved analytical techniques become available and interest in this field increases, the influence of pharmaceuticals on the environment is increasingly being documented. Identified cases where pharmaceuticals are causing detrimental effects are therefore becoming more common. For example, the veterinary use of the non-steroidal anti-inflammatory drug diclofenac has recently been linked to widespread (over 90%) declines in vulture species in some countries^14^ and serves as a reminder that, sometimes, the effects of biologically active drugs can have far-reaching consequences.

While the potential for pharmaceuticals to lead to toxicity in non-target organisms exists across all therapeutic groups, the case of psychotropic medication is particularly concerning. This is because these drugs, which are among the most commonly detected in aquatic environments, affect not only the central nervous system but are also linked with reproduction, growth and immune functions.^13^^,^^15^ In this way, psychotropic drugs such as antidepressants modulate neurotransmitters serotonin, dopamine and noradrenaline, having multiple physiological effects in humans such as weight gain, fatigue and sexual dysfunction.

The ability of psychotropic medication to disrupt the normal biological systems of abundant and ecologically important groups of non-target organisms in aquatic environments is extensive. Since reuptake transporters and receptors evolved in invertebrates such as molluscs and Crustacea, release of neurohormones would be expected to have multiple biological effects on these invertebrates, in addition to vertebrates such as fishes. Therefore, any compounds in the environment at a sufficient concentration able to alter neurohormones have the capacity to affect a wide range of biological processes,^16^ leading to salient effects on critical life cycle events. Processes affected include reproduction, growth, maturation, metabolism, immunity, feeding, locomotion, colour physiology and behaviour. Examples of adverse effects that have been observed include photo- and geotactic behaviour, abnormal activity patterns, aggressive behaviour, developmental and metabolic abnormalities, and reproductive abnormalities. Interestingly, some of these effects are only exhibited at low concentrations, rather than being dose dependent as may have been expected.^16^

For example, Guler and Ford^17^ studied the effects of a variety of pharmaceuticals and the hormone serotonin on the preference for light versus dark choice chambers in amphipods. They reported a significant preference for light and response to gravity in terms of position in amphipods exposed to fluoxetine and serotonin. The dose response was linear for serotonin, whereas for fluoxetine the response was not dose dependent and the behaviour was induced only at lower concentrations (10–100 ng/L). Similarly, when crabs were injected with serotonin, photonegative behaviour was reduced and they spent substantially less time hidden.^16^ This would have consequences for aquatic life, as preference to light has been demonstrated to increase the likelihood of predation.

Other studies found that while 1 ng/L fluoxetine influenced learning in the cuttlefish, 100 ng/L did not, but did significantly influence the retention of memory. Effects observed include fluoxetine influencing on learning and memory in cuttlefishes at concentrations between 1 and 100 ng/L.^18^ Swimming activity has been observed to be altered in amphipod crustaceans at levels as low as 1–100 ng/L,^19^ and gonadal aberrations in zebra mussels have been induced in ranges as low as 20 ng/L. Moreover, fluvoxamine was found to induce egg deposition in zebra mussels at ~318 ng/L,^20^ and exposure to venlafaxine has caused foot detachment (an inability to cling to the side of a tank) at levels as low as 313 pg/L and 31.3 ng/L.^21^^,^^22^ Further effects on reproductive output in terms of frequency of broods, offspring production, gamete release and gene expression have been demonstrated in the ng/L concentrations.

Induction of hyperglycaemic responses in a variety of crustaceans has also been observed. The regulation of blood glucose through crustacean hyperglycaemic hormone is under the control of a variety of neurohormones.^23^ There have also been reports that dopamine, serotonin, noradrenaline and adrenaline are all effective in inducing hyperglycaemic responses in a variety of crustaceans.^24^ The release of crustacean hyperglycaemic hormone has been shown to be promoted by injection with serotonin in a variety of species.^23^ Interestingly, studies with crabs have shown that both serotonin and fluoxetine can stimulate crustacean hyperglycaemic hormone and suppress moulting hormones.^25^ In terms of pigmentation, serotonin has been shown to influence red pigment-dispersing hormone, while dopamine influences red and black pigment-concentrating hormones in shrimp. It has also been found that noradrenaline triggers release of black pigment-dispersing hormone.^23^ Therefore, any drugs with the capability to modulate these neurohormones can conceivably interfere with the camouflage abilities of aquatic invertebrates.

Perhaps it is not surprising that fish exposed to antidepressants or antianxiety medication display altered behaviours when one considers how evolutionarily conserved the nervous system is among the vertebrates. Furthermore, because of this conserved ancestry, fish are now more commonly used in drug discovery, with behavioural tests commonly used in rats and mice being translated to fish models.^26^^,^^27^ For example, the novel tank test measures the ‘normal’ reluctance of a fish to venture in the open surroundings of a new tank, which can be altered by antianxiety medication.^28^^,^^29^ Other studies within the field of environmental toxicology have observed a wide range of altered behaviours in fish, including aggression towards conspecifics,^30^^,^^31^ reproduction,^32^^,^^33^ predator avoidance^34^ and feeding.^35^

A body of evidence is therefore building which suggests that antidepressants in particular, at concentrations found in surface, waste and ground waters, can cause a wide variety of effects. Whether these are occurring in the field downstream of wastewater treatment plants (WWTPs) represents an important and challenging question to address, as the ability to measure abnormal behaviour *in situ* remains a logistical and technological challenge. Moreover, while the underlying role of neurotransmitters has been described in vertebrates, there is considerable paucity of data on their role in invertebrates.^16^ The non-monotonic dose responses shown by some drugs,^17^^,^^18^^,^^36^^–^^38^ for which pharmacological effects are not dose dependent and a response is triggered by a low concentration, with no response to higher concentrations, poses questions which are difficult to ignore.^39^ While some studies have been conducted on the toxicology of antidepressants such as fluoxetine,^16^ these are few and far between. Given the evidence on the influence of pharmaceuticals, particularly psychotropic drugs, on the environment, it can be argued that greater emphasis should be placed on how they may be affecting aquatic life.

## The solutions

The solutions to these problems, as might be expected, are multifactorial and somewhat influenced by historical decisions. For example, some WWTPs may have historically serviced small towns and villages which later grew in population into large towns and cities. If these WWTPs historically discarded their effluent into small (low flow) rivers, then the effluent to river water ratios could change over time. Changes in water usage upstream, for example, water extraction for farming, could further confound the problem. Advanced treatment at wastewater treatment facilities would reduce many of the potentially harmful pharmaceuticals waste products and their breakdown metabolites. Where technological improvements of sewage treatment have been implemented, there have been reductions in intersex (feminised) fish caused by steroid oestrogens and their mimics, as well as improvements in river biodiversity.^40^^,^^41^ These improvements, however, are costly. Owen and Jobling (2012)^42^ estimated that upgrading all the WWTPs in England and Wales to comply with EU regulation to bring synthetic oestrogens below an average of 0.035 pg/L ethinylestradiol per annum would cost an estimated £26 billion.

Another solution to this complex problem is to change behaviours whereby people would traditionally flush their unwanted medications down the toilet or dispose of them in the bin. ‘Take back’ programmes vary in their popularity across Europe, but serve as one means to prevent unwanted medication entering aquatic systems directly following wastewater treatment or indirectly through underground seepage from landfills. The question of green pharmacy has also been raised, whereby the pharmaceutical industry considers the cradle-to-grave approach of their products and designs drugs which readily break down. However, this is extremely difficult to achieve for most drugs, owing to the need to produce pharmacologically active pharmaceuticals in suitable formulations.^43^

## Reflections for policy and practice

Mental healthcare services are provided through complex systems, which are generally based around the use of medication, with training in psychiatry covering vast areas to enable the provision of quality care to patients. However, there is no inclusion of aspects of pollution and the effects of psychotropic medication, and how this could affect aquatic environments. Could educating the medical profession help improve the utility of take back programmes and patient behaviour with regard to drug waste? These substances are not currently covered by existing regulations with regards to sewage management, and analytical methods for detection are just now becoming available. Adequate resources for the diagnosis and management of mental health conditions could help reduce the need for medication and the documented toxic effects of the use of these drugs on non-target organisms. ‘Prescribing’ psychotropic medication for our rivers and estuaries poses a potential risk to aquatic life. Further knowledge and education on adequate therapeutic choices, and improved resources for diagnosis, could support prescribers and practitioners to make environmentally sensible choices, based on evidence of efficacy and safety.^44^

## About the authors

**Alex T. Ford** is a professor of Biology in the Institute of Marine Sciences, School of Biological Sciences, University of Portsmouth, UK; **Helena Herrera** is a senior lecturer in Pharmacy Practice at the School of Pharmacy and Biomedical Sciences, University of Portsmouth, UK.
